# Reduction of the fluorine-18-labeled fluorodeoxyglucose dose for clinically dedicated breast positron emission tomography

**DOI:** 10.1186/s40658-019-0256-9

**Published:** 2019-11-29

**Authors:** Yoko Satoh, Tetsuro Sekine, Yoshie Omiya, Hiroshi Onishi, Utaroh Motosugi

**Affiliations:** 1 Yamanashi PET Imaging Clinic, Shimokato 3046-2, Chuo City, Yamanashi Prefecture 409-3821 Japan; 2 Department of Radiology, Nippon Medical School, Bunkyo-ku, Tokyo, Japan; 3 Department of Radiology, University of Yamanashi, Chuo City, Yamanashi Prefecture Japan

**Keywords:** Breast cancer, Dedicated breast positron emission tomography, Fluorodeoxyglucose, Dose reduction

## Abstract

**Purpose:**

To determine the clinically acceptable level of reduction in the injected fluorine-18 (^18^F)-labeled fluorodeoxyglucose (^18^F-FDG) dose in dedicated breast positron emission tomography (dbPET).

**Methods:**

A breast phantom with four spheres exhibiting various diameters (5, 7.5, 10, and 16 mm), a background ^18^F-FDG radioactivity of 2.28 kBq/mL, and a sphere-to-background radioactivity ratio of 8:1 was used. True dose-reduced dbPET images were obtained by data acquisition for 20 min in list mode at multiple time points over 7 h of radioactive decay. Simulated dose-reduced images were generated by reconstruction with a portion of the list mode acquisition data. True and simulated dose-reduced images were visually and quantitatively compared. On the basis of the phantom study, dbPET images for 32 breasts of 28 women with abnormal uptake were generated after simulated reduction of the injected ^18^F-FDG doses; these images were compared with those acquired using current clinical doses.

**Results:**

There were no qualitative differences between true and simulated dose-reduced phantom images. The phantom study revealed that the minimal required dose was 12.5% for the detection of 5-mm spheres and 25% for precise semi-quantification of FDG in the spheres. The 7-min reconstruction with a 100% dose was defined as the reference for the clinical study. The image quality and lesion conspicuity were clinically acceptable for the 25% dose images. Lesion detectability on the 12.5% dose images was maintained despite image quality degradation.

**Conclusions:**

In summary, 25% of the standard ^18^F-FDG dose for dbPET can provide a clinically acceptable image quality, while 12.5% of the standard dose results in acceptable quality in terms of lesion detection when lesions are located at a sufficient distance from the edge of the dbPET detector.

## Introduction

Fluorine-18 (^18^F)-labeled fluorodeoxyglucose (FDG) positron emission tomography (PET)/computed tomography (CT) has become one of the most useful tools for the diagnostic imaging of malignancies, including breast cancer. It is also used for staging or re-staging, monitoring of treatment responses, and prognostic predictions [1–3]. However, with regard to breast cancer, FDG PET is mainly used for the detection of metastasis or recurrence because of its limited spatial resolution. Therefore, it has been challenging to detect small breast cancers using whole-body PET/CT [4].

High-resolution dedicated breast PET (dbPET) scanners have been developed to detect small breast lesions. High-resolution dbPET involves either positron emission mammography (PEM) or a tomographic technique using a ring-shaped scanner [5]. These dbPET systems have greater spatial resolution and sensitivity than does whole-body PET/CT [6]. Combining dbPET and PET/CT enables local and systemic assessment of breast cancer in a single examination session without additional exposure to PET/CT, because dbPET does not use additional X-rays for attenuation correction, which is performed via extraction of a breast contour from acquisition data as internal homogenous fat.

PET/CT results in considerable radiation exposure (approximately 7–10 mSv in recent studies, derived from the injected dose of radiotracer and X-ray CT used for attenuation correction and anatomic co-registration) [7, 8]. Exposure reduction is a major concern in PET, and several studies have reported on dose reduction of 18F-FDG in PET/CT [9, 10] or PET/magnetic resonance (PET/MR) [11–13]. By combining long acquisition (20 min) of breast regions on PET/MR system with high sensitivity detector consisting of silicon photomultiplier, the radiation exposure was successfully reduced comparable to the effective dose of a single digital mammogram [13]. It is expected that a similar dose reduction would be achieved by dbPET which system can omit radiation exposure for attenuation correction and consists of a high sensitivity detector. When dbPET is used alone, the exposure dose is half that of PET/CT because it does not use X-ray CT for attenuation correction. In addition, because dbPET can achieve high resolution and sensitivity by its four-layer depth-of-interaction detector [14], it may allow comparable or further reduction of the injection dose relative to that used in PET/MRI. However, reports on dose reduction in dbPET are lacking. The purpose of the present study was to determine the clinically acceptable level of reduction in the injected ^18^F-FDG dose for dbPET.

## Methods

### Ring-shaped dbPET scanner

The ring-shaped dbPET scanner (Elmammo, Shimadzu Corp., Kyoto, Japan) consisted of 36 detector modules arranged in three contiguous rings, with a diameter of 195 mm, axial length of 156.5 mm, and depth-of-interaction measurement capability [15]. The transaxial effective field-of-view (FOV) was 185 × 156.5 mm^2^. Each detector block consisted of a four-layered 32 × 32 array of lutetium oxyorthosilicate crystals coupled to a 64-channel positron-sensitive photomultiplier tube via a light guide. Performance metrics included 1.5-mm FWHM resolution in standard mode in the axial, sagittal, and coronal views; detector sensitivity of 0.09–0.13 cps/Bq at the center of the detector; and detector sensitivity of 0.05–0.08 cps/Bq at a quarter depth of the detector. The peak noise equivalent count was 600–800 kcps.

### Development and preparation of the breast phantom

**Fig. 1:**
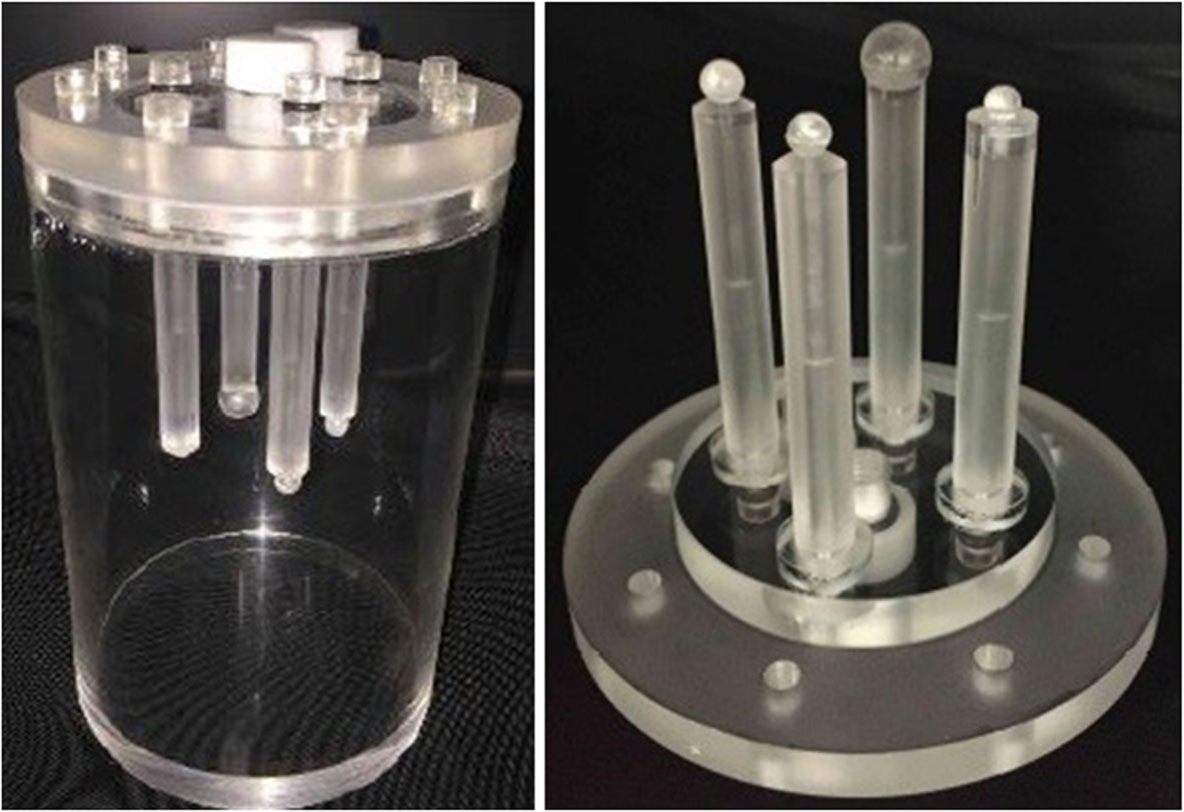
A cylindrical breast phantom with four spheres of various diameters (5, 7.5, 10, and 16 mm)

We acquired dbPET images using a cylindrical breast phantom with four spheres of various diameters (5, 7.5, 10, and 16 mm [Fig. 1]). The spheres and background were filled with ^18^F-FDG solution. The background radioactivity was 2.28 kBq/mL, and the sphere-to-background radioactivity ratios (SBRs) were 8:1.

### Phantom data acquisition and image reconstruction

The phantom was placed at the center of the column in the dbPET such that the four spheres were aligned horizontally and then scanned for 20 min in list mode under the following various conditions.

The dbPET images were reconstructed using a three-dimensional list mode dynamic row-action maximum-likelihood algorithm with one iteration and 128 subsets, a relaxation control parameter of *β* = 20, a matrix size in the axial view of 236 × 200 × 236 with a post-reconstruction smoothing Gaussian filter (1.17 mm FWHM), and scatter correction. Attenuation correction was calculated using a uniform attenuation map with object boundaries obtained from emission data [16], and scatter corrections were applied for all images. Scatter correction was performed using was the convolution-subtraction method [17] with kernels obtained by background tail fitting.

### True and simulated ^18^F-FDG dose reduction of dbPET images

First, for the true dose reduction, dbPET images were acquired at a fixed position for 20 min in list mode every 55 min (a half of ^18^F) until 440 min later. Due to the steady decay of the phantom activity, these measurements were equivalent to reduced doses (tracer dose reduction of 50% after each half-time).

**Fig. 2:**
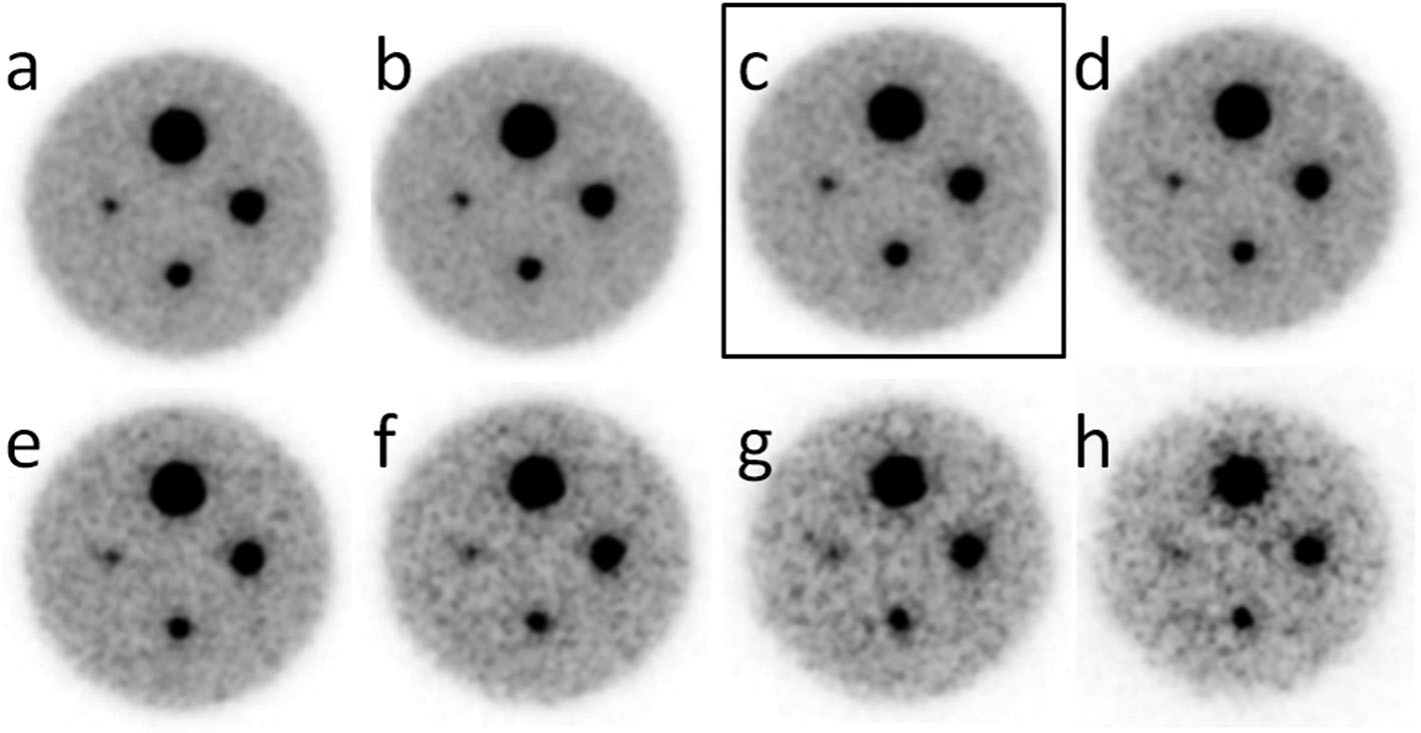
Phantom images with different acquisition times created to determine the optimal acquisition time as a reference: **a** 840 s (200% dose), **b** 630 s (150% dose), **c** 420 s (100% dose), **d** 315 s (75% dose), **e** 210 s (50% dose), **f** 105 s (25% dose), **g** 52 s (12.5%), and **h** 26 s (6.25% dose). The numbers represent the acquisition time. Percentages in parentheses indicate the percentage of a 420-s acquisition (surrounded by a rectangle) as 100%

Second, the simulated dose-reduced images were obtained using dbPET data of various acquisition times (1050, 840, 240, 315, 210, 105, 52, and 26 s) at the beginning of the list mode data of each dose acquisition (Fig. 2a).

### Phantom image analysis

The maximum standardized uptake value (SUV_max_) and the mean standardized uptake value (SUV_mean_) of each sphere and the background were measured on all phantom images using Pmod software (Ver. 4.0, PMOD Technologies LLC, Zurich, Switzerland). The volume-of-interest (VOI) for each sphere was the sum of the volumes of the voxels with SUVs that were greater than or equal to 40% of their SUVmax. The 20 VOIs of 5 mm in diameter for the background were placed on the same axial plane with hot spheres and ± 20 mm slices. The SUV_max_ and SUV_mean_ were the average values using a total of 100 VOIs.

### Patient data acquisition and image reconstruction

After fasting for at least 6 h, patients received ^18^F-FDG (3 MBq/kg), and dbPET scanning was performed for 7 min for each breast 90 min after injection of ^18^F-FDG. The dbPET image reconstructed with 7 min acquisition data was defined as the standard (100% of injected ^18^F-FDG dose). The simulated dose-reduced images were obtained using the dbPET data of various acquisition times (from 420 to 26 s) at the beginning of the list mode data. Reduction of injected ^18^F-FDG was simulated by five other different time sets: a reconstruction followed by reconstructions simulating 50% (210 s), 25% (105 s), 12.5% (57 s), and 6.25% (26 s) of the original dose.

### Evaluation of clinical images and statistical analysis

A total 165 reconstructed dbPET data sets (33 breasts of 29 women with five different reconstructions) were evaluated separately by two experienced nuclear medicine physicians (with 14 and 7 years of experience interpreting PET, respectively). These readers were blinded to the clinical background, reconstructed set, and simulated injected ^18^F-FDG dose. PET-data sets were viewed in transaxial and sagittal planes, and medio-lateral maximum intensity projection (ML-MIP) images. The two readers assessed the image quality and conspicuity of the abnormal uptake which was indicated on the reference image (100% dose) as a reference to the standard images in random order using a four-point scale as follows: (0) poor image quality, not diagnostic; (1) decrease in image quality, loss of diagnostic ability concerned; (2) slight decrease in image quality, no clinical problem; and (3) equivalent to reference. A score of 2 or higher was defined as clinically acceptable, and a score of 1 or higher was also considered acceptable to detect the target lesions. Inter-observer reproducibility was evaluated by kappa-statistics. Regarding visual assessment of clinical dbPET images, comparison of different reconstruction settings was performed using the Mann-Whitney *U* test.

The SUV_max_ of mass-like uptakes were also evaluated. The VOI was defined as a volume with 40% or more SUV_max_ of that VOI on the dbPET image at 100% ^18^F-FDG dose. The VOI was copied and pasted onto the other dose-reduced images. Except for mass-like uptake, focus and non-mass uptake were excluded because their quantitative reliability could not be established.

This single-institution retrospective study was approved by our institutional review board. The board waived the requirement for written informed consent from the patients.

## Results

### dbPET phantom studies

Using the 20-min list mode acquisition data from the first scan, which was the full injection dose, the phantom images with different acquisition times were first created to determine the optimal acquisition time as a reference. Based on these dose reduction images, 7 min (420 s) acquisition was defined as the standard acquisition time in this study (Fig. 2).

**Fig. 3:**
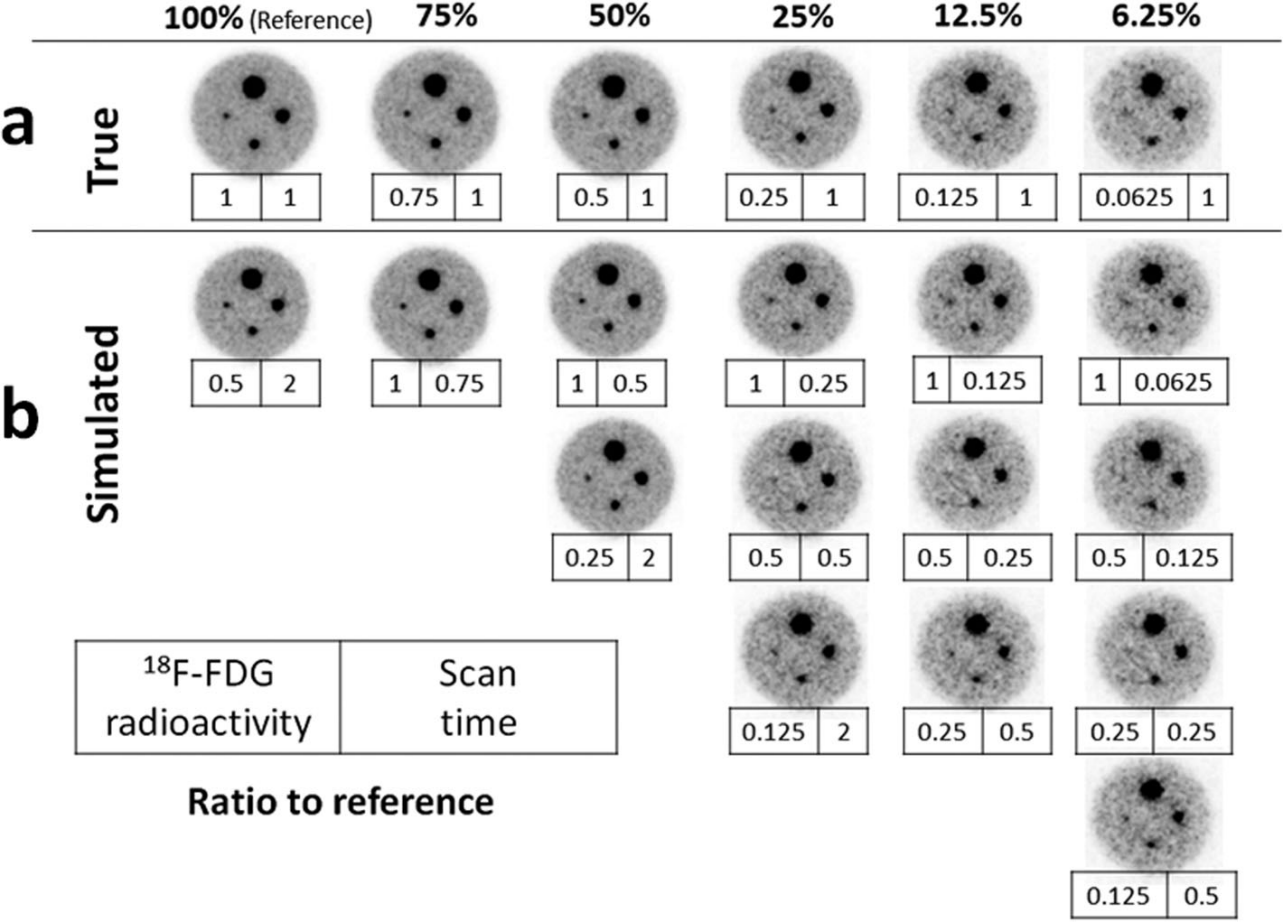
**a** dbPET phantom images acquired with true dose reduction, with 75%, 50%, 25%, 12.5%, and 6.25% of the ^18^F-FDG radioactivity relative to that in the first scan. **b** Images acquired with simulated dose reduction. The numbers in the frame attached to each image show the rate of ^18^F-FDG radioactivity (left in the frame) and acquisition time (right in the frame) relative to that in the first scan. On the basis of these dose reduction images, 7 min (420 s) was defined as the standard acquisition time

The quality of simulated dose reduction phantom images was the same as that of true dose reduction images. Focusing on the smallest 5 mm sphere, it was visible down to a dose of 25%. At lower doses, it was difficult to distinguish hot spheres from the background at SBR of 8:1 (Fig. 3).

Additional file 1: Figure S1 shows the SUV_max_ (A) and SUV_mean_ (B) of four spheres, and the background could be evaluated equally down to the 25% dose. The average and variability of SUV_max_, SUV_mean_ of each hot sphere, and the background at true and simulated 25% injection doses were as follows. SUV_max_: 16 mm, 10.5 ± 2.6%; 10 mm, 11.3 ± 7.2%; 7.5 mm, − 5.7 ± 5.5%, 5 mm, − 14.4 ± 7.6%; background 23.0 ± 0.4%; SUV_mean_: 16 mm, − 3.1 ± 0.7%; 10 mm, − 6.4 ± 1.2%; 7.5 mm, − 11.6 ± 2.6%; 5 mm, − 11.9 ± 3.7%; background − 0.9 ± 1.2%, respectively. SUV_max_ was overestimated at less than the 25% dose.

### Patient studies

A total of 32 breasts with one or more abnormal FDG uptakes on dbPET of 28 women were evaluated. Of these 32 breasts, 20 (62.5%) were diagnosed as “cancer,” five (15.6%) were “non-malignant,” and seven (21.9%) were “unknown.” These were diagnosed based on histopathological findings and clinical follow-up.

**Fig. 4:**
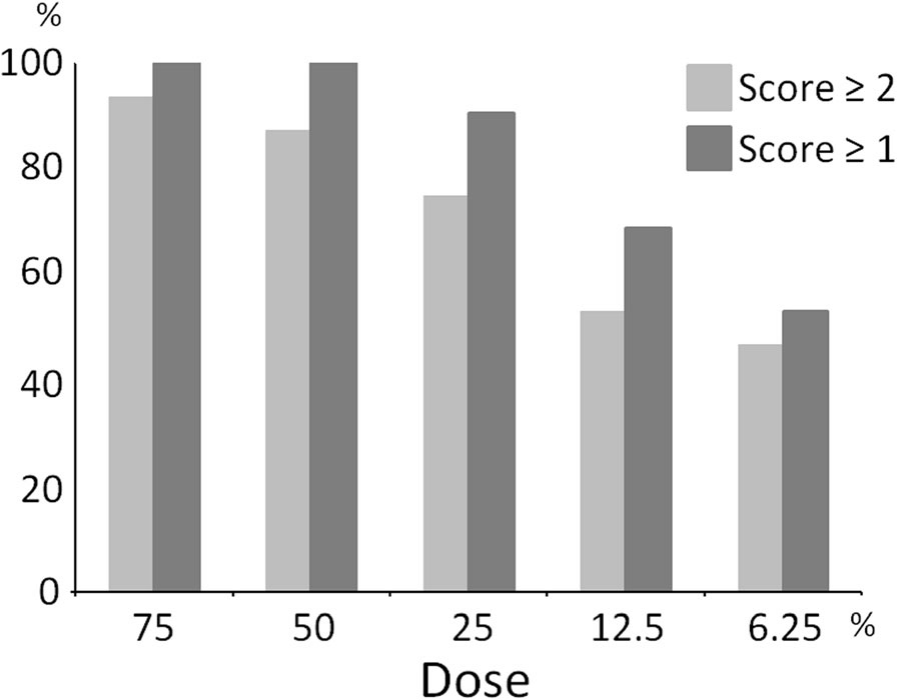
Bar chart showing the percentage distribution of the image quality for dbPET images acquired at five reduced doses. Abbreviation: dbPET, dedicated breast positron emission tomography

**Fig. 5:**
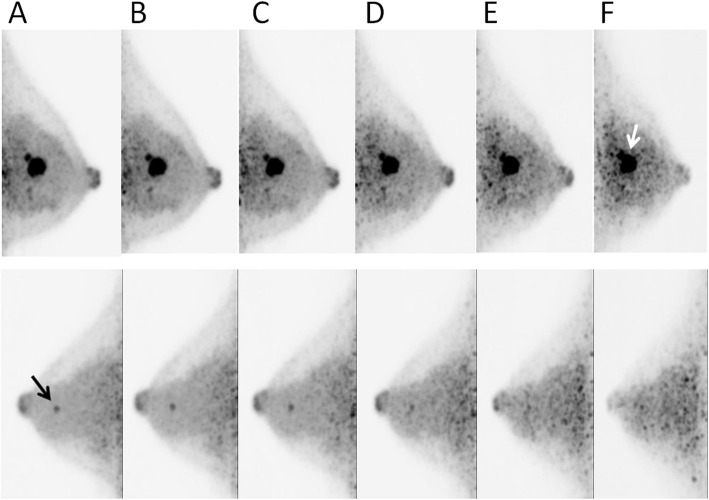
A representative case involving a 44-year-old patient with bilateral breast cancers: **a** 100% (acquisition time was 420 s), **b** 75% dose (acquisition time was 315 s), **c** 50% dose (acquisition time was 210 s), **d** 25% dose (acquisition time was 105 s), **e** 12.5% dose (acquisition time was 52 s), and **f** 6.25% dose (acquisition time was 26 s) of the simulated ^18^F-FDG dose. The left-sided breast cancer of 13 mm in diameter (white arrow) was confirmed with 6.25% dose as well as that for 100% dose (upper row). The right-sided breast cancer of 6 mm in diameter (black arrow) was unclear and difficult to distinguish from noise at the 12.5% dose or lower. Abbreviation: ^18^F-FDG, fluorine-18-labeled fluorodeoxyglucose

Inter-observer reproducibility was good (kappa-value = 0.69). The scores evaluated by one reader with longer experience were analyzed. For clinical imaging, 75% and 50% dose reduction imaging seemed adequate. In the 25% dose images, the image quality and conspicuity of the lesion scores were 2 or higher in 75% of the breasts. In the 12.5% dose images, the score was ≥ 1 for 68.8% breasts (Table 1, Fig. 4). A representative case is shown in Fig. 5.

**Table 1:** Image quality score for dose-reduced dbPET images of 32 breasts

	Simulated injection dose				
	75%	50%	25%	12.5%	6.25%
Mean	2.69	2.34	2.06	1.47	1.22
SD	0.59	0.7	1.01	1.19	1.26
≥ Score 2	30/32 (93.8%)	28/32 (87.5%)	24/32 (75%)	17/32 (53.1%)	15/32 (46.9%)
≥ Score 1	32/32 (100%)	32/32 (100%)	29/32 (90.6%)	22/32 (68.8%)	17/32 (53.1%)

SUV_max_ and its percentage difference for the 20 lesions with mass-like uptake were also compared among the reconstructed images (Additional file 2: Figure S2). SUV_max_ was maintained or increased for most lesions. In addition, three of the 20 lesions exhibited a significant decrease in SUV_max_ at lower doses. These were small in size and located near the chest wall.

## Discussion

In the current study, reconstructed phantom images with different acquisition times were assessed for the determination of clinically optimal dbPET parameters. The spheres were visualized equally when they were acquired over ≥ 5 min; however, the background was smoother when it was acquired over 7 min than when it was acquired over 5 min. Since the scan time of 7 min on one side and 14 min on bilateral breasts was also considered to be clinically tolerable, the standard acquisition time was determined as 7 min in this study. In previous clinical studies using dbPET, 5-min acquisition for a breast is common [18]. Based on our current phantom and clinical study, there was no difference between the 5- and 7-min acquisition times in the ability to visualize lesions, but the background was smoother on images with 7-min acquisition than on those with 5-min acquisition. Image quality improves with longer acquisition times if the conditions of the facilities allow. The simulated dose reduction images were then reconstructed from the list mode data obtained with several ^18^F-FDG doses and compared to true dose-reduced images regarding ^18^F-FDG radioactivity concentration-acquisition time. As shown in the results, there was no visual difference between the true and simulated dose-reduced images. This indicates that the simulated dose-reduced clinical images reconstructed with the same method as that in this phantom study could be considered visually equivalent to true dose-reduced images. In the quantitative evaluation of the phantom study, the variations in SUV_max_ and SUV_mean_ of the hot spheres and background were acceptably small at 25% or higher dose. This suggests that even when the dose is reduced to 25%, a quantitatively comparable image can be obtained. However, SUV_max_ was overestimated with larger spheres at lower doses than 25%. This is considered to be a severe partial-volume effect in small spheres. These results were consistent with the previous phantom study of PET/CT on ^18^F-FDG dose reduction [10]. Furthermore, the SUV_max_ and SUV_mean_ were underestimated at very low dose of about 5% or less, possibly due to excessive scatter correction.

Our clinical study using simulated dose-reduced images of dbPET showed that the image quality was clinically acceptable with 25% of the standard injected ^18^F-FDG dose (reduction of up to 75%). Furthermore, the overall image quality with 12.5% of the standard injected ^18^F-FDG (reduction of up to 87.5%) dose decreased considerably; however, it was acceptable for simple lesion visualization. In the quantitative assessment of mass-like uptakes, SUV_max_ of most lesions were either overestimated or not changed with low dose, whereas SUV_max_ of the three small lesions close to the chest wall were significantly decreased at lower doses. This was considered to be due to the deterioration of image quality close to the detector’s edge and excessive scatter correction at the very low injected dose.

When using dbPET repeatedly, such as in screening of a high-risk group for breast cancer or re-imaging of abnormal uptake that can be confirmed only by dbPET and is not detected by other modalities, it is not always necessary to employ concurrent whole-body PET/CT. Recently, the American College of Radiology and Japan Association of Breast Cancer Screening recommended breast cancer screening using a contrast-enhanced MRI for high risk groups [19, 20]. It has been reported that PEM has equal diagnostic ability as that of contrast-enhanced MRI [21]. and is useful for breast cancer screening [22–24]. Therefore, dbPET is considered useful to screen high-risk groups. A reduction of 75% injected ^18^F-FDG dose would result in an estimated effective dose of approximately 0.9 mSv for a patient weighting 60 kg (3 MBq/kg × 25/100 × 60 kg × 0.0199 mSv/MBq) [25]. The exposure of PET/CT is about 20 times that of mammography which is used for breast cancer screening, while the exposure of dbPET with dose reduction can be reduced to about twice. Furthermore, radiation burden in dbPET accounts for the whole body but is focused on a breast in mammography, and the radiation burden to other organs is less than 1% of the focused breast [26]. Despite these differences, low-dose dbPET will be a powerful tool that can routinely be used in breast cancer care.

We demonstrated that the simulated dose-reduced images derived from the clinical PET data were reliable by comparing them with true dose-reduced phantom images. In recent years, referred as ultra-low-dose PET, machine learning approaches have been applied to reduce the radiotracer dose in the PET imaging [27]. Chen et al. has been reported that PET/MR combined with deep learning enabled it [28]. Especially recently, the U.S. Food and Drug Administration has approved to apply innovative deep learning imaging technologies to improve image quality of low-dose PET data (SubtlePET https://subtlemedical.com/subtle-medical-receives-fda-510k-clearance-and-ce-mark-approval-for-subtlepet/). The machine learning approach is expected to be able to achieve further dose-reduction in dbPET imaging.

There were several limitations in our current study. First, a true dose-reduced study could not be performed on clinical images from an ethical point of view. Although supported by the phantom study, there may be various differences between the phantom and humans. Second, the simulated dose-reduced images were obtained using PET data with various acquisition times of the first frame after segmentation due to the limitation of the software attached to the device. In clinical imaging, it is preferable to collect data randomly from the whole data for the reconstruction of simulated dose-reduced images to avoid the effects of body movement during acquisition. Third, although there was a difference in quantitative evaluation depending on the location of the lesions in the clinical study, the sample size was small.

In conclusion, 25% of the standard dose of ^18^F-FDG (reduction of up to 75%) for dbPET can result in clinically acceptable image quality. Moreover, 12.5% of the standard dose of ^18^F-FDG (reduction of up to 87.5%) can result in acceptable image quality for lesion detection, with the exception of small lesions located close to the edges of the dbPET detector.

## Supplementary information

**Additional file 1: Figure S1.** The SUV_max_ (A) and SUV_mean_ (B) of four spheres and the background. Impact of ^18^F-FDG dose on SUV_max_ (A) and SUV_mean_ (B) in four spheres of different diameter. Abbreviations: ^18^F-FDG, fluorine-18-labelled fluorodeoxyglucose; SUV_max_, maximum standardized uptake value; SUV_mean_, mean standardized uptake value.

**Additional file 2: Figure S2.** Impact of simulated reduced injected dose of ^18^F-FDG on SUV_max_ of all lesions with mass-like uptake on clinical images. Simulated injection dose on x-axis correspond to 100, 75, 50, 25, 12.5, and 6.5% dose from the left. The percentage of SUV_max_ based on that of full dose on y-axis (A). The volume (cm^3^) of each mass-like uptake on x-axis was the sum of voxels with 40% SUVs that were greater than or equal 40% of its SUV_max._ The percentage of SUV_max_ based on that of full dose on y-axis (B).

## Data Availability

The datasets used and/or analyzed during the current study are available from the corresponding author on reasonable request.

## Abbreviations

^18^F-FDG: Fluorine-18-labeled fluorodeoxyglucose
dbPET: Dedicated breast positron emission tomography
