# Supplementary figures and images for: Reduction of the fluorine-18-labeled fluorodeoxyglucose dose for clinically dedicated breast positron emission tomography
Source: EJNMMI Phys. 2019 Nov 29;6:21. doi: 10.1186/s40658-019-0256-9 (PMC6884607; doi:10.1186/s40658-019-0256-9)

A


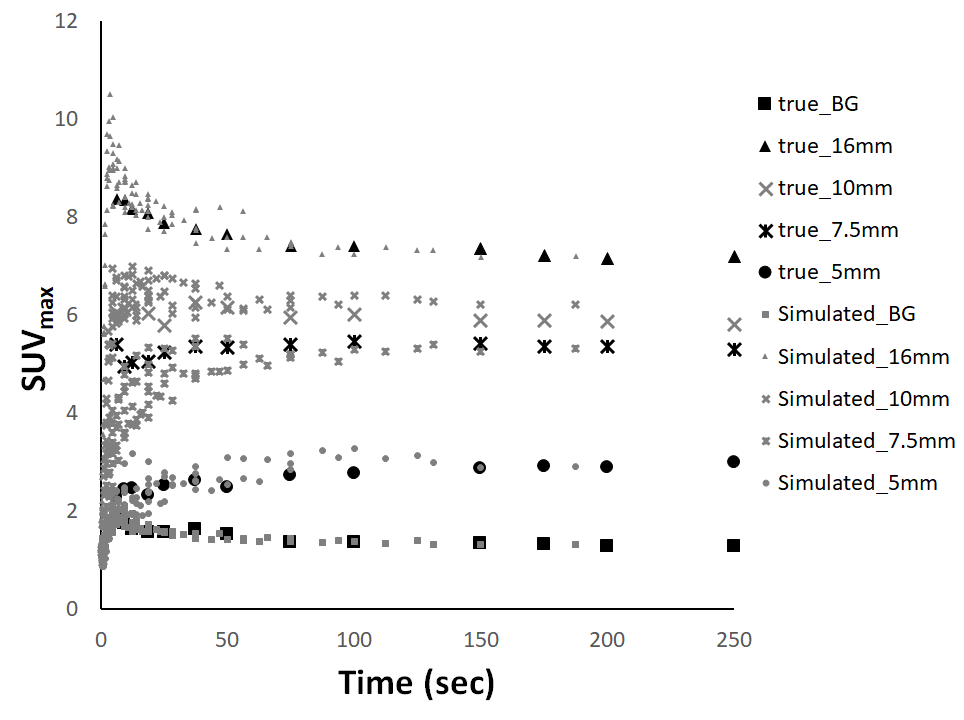


B.


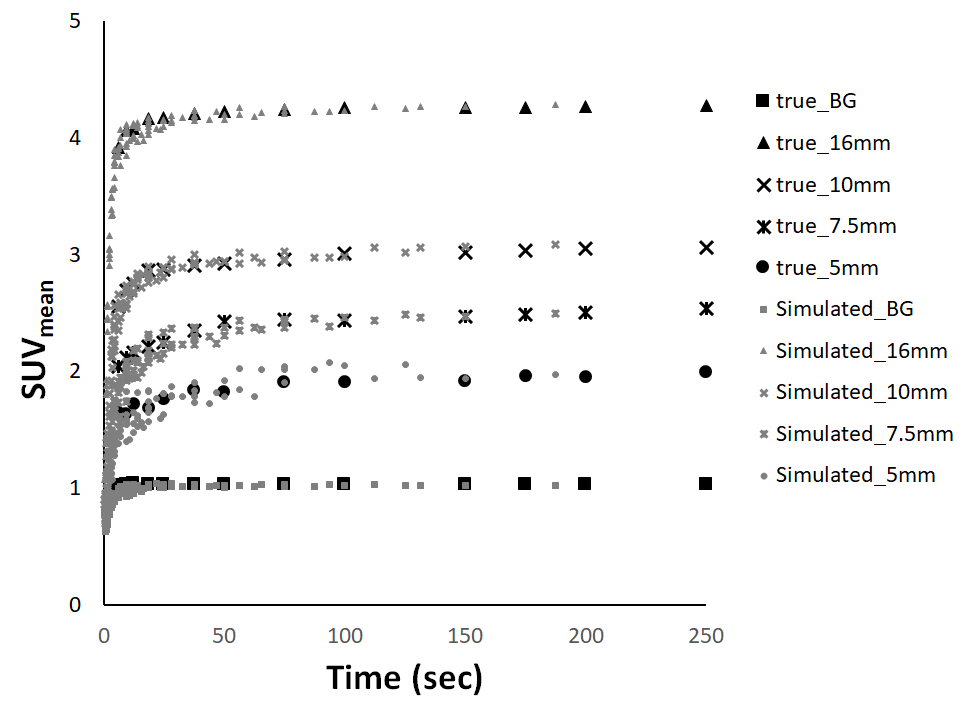

Supplement: Supplementary file 1 — Additional file 1: Figure S1. The SUVmax (A) and SUVmean (B) of four spheres and the background. Impact of 18F-FDG dose on SUVmax (A) and SUVmean (B) in four spheres of different diameter. Abbreviations: 18F-FDG, fluorine-18-labelled fluorodeoxyglucose; SUVmax, maximum standardized uptake value; SUVmean, mean standardized uptake value. [file 40658_2019_256_MOESM1_ESM.docx]

A


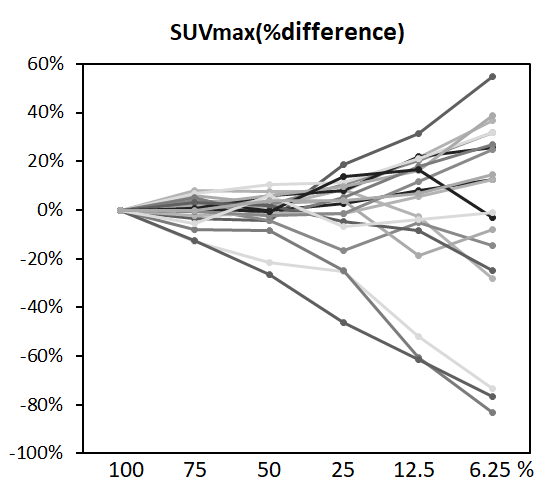


B


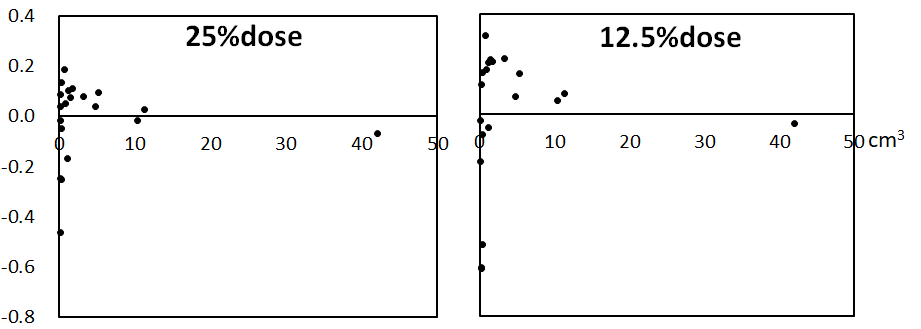

Supplement: Supplementary file 2 — Additional file 2: Figure S2. Impact of simulated reduced injected dose of 18F-FDG on SUVmax of all lesions with mass-like uptake on clinical images. Simulated injection dose on x-axis correspond to 100, 75, 50, 25, 12.5, and 6.5% dose from the left. The percentage of SUVmax based on that of full dose on y-axis (A). The volume (cm3) of each mass-like uptake on x-axis was the sum of voxels with 40% SUVs that were greater than or equal 40% of its SUVmax. The percentage of SUVmax based on that of full dose on y-axis (B). [file 40658_2019_256_MOESM2_ESM.docx]
